# Metabolic Associated Fatty Liver Disease in Children—From Atomistic to Holistic

**DOI:** 10.3390/biomedicines9121866

**Published:** 2021-12-09

**Authors:** Cristina Oana Mărginean, Lorena Elena Meliț, Maria Oana Săsăran

**Affiliations:** 1 Department of Pediatrics I, George Emil Palade University of Medicine, Pharmacy, Science, and Technology of Târgu Mureș, Gheorghe Marinescu Street No 38, 540136 Târgu Mureș, Romania; marginean.oana@gmail.com; 2 Department of Pediatrics III, George Emil Palade University of Medicine, Pharmacy, Science, and Technology of Târgu Mureș, Gheorghe Marinescu Street No 38, 540136 Târgu Mureș, Romania; oanam93@yahoo.com

**Keywords:** children, obesity, MAFLD

## Abstract

Non-alcoholic fatty liver disease has become the most common chronic liver disease in children due to the alarmingly increasing incidence of pediatric obesity. It is well-documented that MAFLD prevalence is directly related to an incremental increase in BMI. The multiple hits theory was designed for providing insights regarding the pathogenesis of steatohepatitis and fibrosis in MAFLD. Recent evidence suggested that the microbiome is a crucial contributor in the pathogenesis of MAFLD. Aside from obesity, the most common risk factors for pediatric MAFLD include male gender, low-birth weight, family history of obesity, MAFLD, insulin resistance, type 2 diabetes mellitus, obstructive sleep apnea, and polycystic ovarium syndrome. Usually, pediatric patients with MAFLD have nonspecific symptoms consisting of fatigue, malaise, or diffuse abdominal pain. A wide spectrum of biomarkers was proposed for the diagnosis of MAFLD and NASH, as well as for quantifying the degree of fibrosis, but liver biopsy remains the key diagnostic and staging tool. Nevertheless, elastography-based methods present promising results in this age group as potential non-invasive replacers for liver biopsy. Despite the lack of current guidelines regarding MAFLD treatment in children, lifestyle intervention was proven to be crucial in the management of these patients.

## 1. Introduction

It is fairly stated that the increase in metabolic associated fatty liver disease (MAFLD), formerly known as non-alcoholic fatty liver disease (NAFLD), incidence is directly related to the increase in obesity incidence worldwide—being known as the most common chronic liver disease in both children and adults from western countries [1]. Children represent a particular age group due to their longer lifespan and associated risk for developing life-threatening complications related to this complex pathology, thereby leading to a considerable burden on healthcare services and the economy. Thus, we must acknowledge the urgent need for the comprehensive and effective management of children with MAFLD. The complex interplay between genetic, epigenetic, and environmental factors represents the key factor in the pathogenesis of MAFLD, which is frequently associated with metabolic syndrome features and type 2 diabetes mellitus [2]. The wide spectrum of MAFLD includes ‘simple steatosis’, which consists of simple fat accumulation within the liver (MAFLD) defined as fat accumulation in >5% of hepatocytes; steatohepatitis (NASH), with or without fibrosis, is characterized by tissue necrotic inflammation, hepatocellular ballooning, and, eventually, the different stages of fibrosis associated with steatosis and cirrhosis. Moreover, the major impact of MAFLD was acknowledged not only on liver-related morbidity and mortality, but also in terms of increased risk of cardiovascular disease, type 2 diabetes mellitus, and mortality during adulthood—representing an important cause of hepatocellular carcinoma [2]. A study performed on 25,000 Danish children proved that childhood obesity is associated with an increased risk for developing hepatocellular carcinoma in adults [3]. Therefore, MAFLD is certainly fueling the increasing incidence and prevalence of liver neoplasia. Furthermore, MAFLD is currently known as the second leading cause for liver transplantation in adults in the USA [4]. Thus, the current global pediatric obesity epidemic foretells of a major increase in MAFLD prevalence and its associated complications, which will haunt the global population for decades to come.

Based on these facts, a holistic approach to MAFLD in children might be the hallmark of successful management strategies.

## 2. Epidemiology

MAFLD is an epidemic global disease affecting individuals of all ages from different ethnic groups such as Europe, the Middle East, and Asia [5,6,7,8,9,10]. Several studies have indicated a prevalence between 3–10% in the general pediatric populations, with a considerable increase of up 70% in individuals with metabolic comorbidities [11]. A recent meta-analysis on MAFLD prevalence in children indicated a higher prevalence in clinically obese populations when compared to general population studies, proving, also, that it predominantly affects males at an incremental increase that is directly related to greater body mass index (BMI) [12]. The same meta-analysis could not reveal a relationship with ethnicity due to the poor information on its distribution in each of the assessed studies [12]. Nevertheless, pediatric studies proved that males and Hispanics of Indigenous American ancestry display a greater risk for developing MAFLD in comparison to females, as well as white and black individuals [13,14]. Moreover, data from the National Health and Nutrition Examination Survey performed on children between 12–19 years of age indicated that 12.4% of the males included in this study had unexplained elevated alanine aminotransferase (ALT) levels in comparison to 3.5% of the females [15]. These variations in MAFLD incidence and prevalence are clearly related to the used diagnostic method. Early studies, which assessed the prevalence of MAFLD in pediatric ages based on aminotransferases levels or ultrasonography, estimated a prevalence ranging between 3–7% [16]. In addition, studies performed in children with obesity proved there was a higher prevalence of elevated ALT, ranging between 8–42%, while the prevalence of ‘bright’ liver at ultrasonography indicated liver steatosis ranged from 1.7–77% [17]. A meta-analysis including studies from 76 different countries underlined an estimate of 7.6% in the general population, and up to 34.2% in obesity clinics [12]. Despite the fact that most of the studies relied on the assessment of ALT and/or imaging techniques such as ultrasonography and magnetic resonance imaging (MRI) to determine the prevalence of MAFLD, these methods were not found to be accurate indicators of fibrosis, since certain patients with mildly increased or normal ALT levels were found to have substantial fibrosis [18]. It was also shown that elevated ALT might be associated with an underestimation of MAFLD in young people with obesity, but it might overestimate this condition in normal-weight subjects [12]. Additionally, ultrasound lacks the sensitivity in identifying steatosis in the setting of macrovesicular fat, which affects less than 30% of hepatocytes [19]. Albeit proton-weighted MRI was found to be accurate and reliable in quantifying liver fat content, its related costs limit the utility in patients with MAFLD [20,21]. Liver biopsy remains the most accurate diagnosis for MAFLD independent of age. Thus, a random autoptic study performed on children who died in accidents in California found a prevalence of histological MAFLD ranging from 0.7 in 2–4 year-old individuals to 17.3% in 15–19 year-old individuals, with an increase of up to 38% in children with obesity [13]. Based on the meta-analysis of Andersen et al., prevalence estimates of MAFLD in clinical studies performed on children and adolescents with obesity, as well as in those from the general population, were significantly lower when using elevated ALT as a diagnostic tool rather than liver biopsy, ultrasound, or MRI [12].

## 3. Pathophysiology

MAFLD pathogenesis is still a poorly understood topic. Nevertheless, fat accumulation within hepatocytes, the first step in the pathogenesis of this condition, is the result of a disbalance between the acquisition of fatty acids and their removal by hepatic oxidation and very low-density lipoproteins (VLDL) secretion [22]. Insulin resistance in adipose tissue has been proved to increase the influx of fatty acids in the liver through lipolysis in adipocytes. Moreover, hyperinsulinemia, as a consequence of insulin resistance, upregulates lipogenesis in the liver. Thus, we can state that insulin resistance represents both a cause and a consequence of hepatic steatosis, and that fatty acids accumulation in the hepatocytes results in insulin signaling pathway defects in individuals with genetic susceptibility [2].

Multiple hits theory explains the pathogenesis of steatohepatitis and fibrosis in MAFLD. The first step consists of fatty acids accumulation in hepatocytes—followed by “lipotoxicity”—while the second hit consists of a complex liver injury caused by the previously mentioned accumulation, leading to inflammation and cell death. In this setting, the clearest lipotoxic insults are mitochondrial dysfunction, endoplasmic reticulum stress, oxidative stress, and perturbation of intracellular signaling pathways [23]. The third hit of this theory describes the consequence of the injury-healing process as a response to the above detailed processes, which results in a recruitment of hepatic progenitor and Kupffer cells that further act as triggers for the activation of the hepatic stellate cells [24]. These cells differentiate into myofibroblasts, which secrete extracellular matrix components and inflammatory cytokines meant to repair the injured hepatic epithelia. However, in the setting of repetitive liver injury, these processes will be overwhelmed and will lead over time to progressive liver fibrosis [23].

Studies performed on adults revealed that the pathophysiology of MAFLD is more complex than described by the multiple hit theory since multiple single nucleotide polymorphisms (SNPs) were proved to be associated to MAFLD, NASH, and fibrosis. Furthermore, large, multi-ethnic cohort studies underlined a major variability between the different populations in terms of MAFLD susceptibility, with the highest risk in Hispanics, followed by Europeans with a moderate risk, and lastly by African Americans, who displayed the lowest risk independent of socio-economic factors, adiposity, or insulin resistance [25]. These differences are mostly related to the well-documented role of genetic factors as major triggers of prevalence variability among ethnic groups [26,27]. Moreover, family studies proved that the family members of individuals with MAFLD-associated cirrhosis have a 12.5-fold higher risk for developing progressive MAFLD as compared to the general population [28]. These statements were further sustained by twin studies that revealed that half of the variability of ALT levels and liver fat content is explained by heritable factors since heritability of hepatic fat and fibrosis are mostly shared [29,30]. Genome-wide studies identified the I148M protein variant of the patatin-like phospholipase domain-containing protein 3 as the most important genetic determinant of the hepatic content, being more frequently encountered in Hispanic individuals [31]. Studies performed on pediatric populations also revealed an increased risk for liver disease in those carrying this variant, which was proved to additionally interact with dietary factors like intake of fructose-enriched drinks [32,33,34]. The I148M variant promotes liver disease by various mechanisms, such as the alteration of both the lipid remodeling and retinol release from hepatic stellate cells [35,36,37,38]. The E167K variant in the transmembrane 6 superfamily member 2 is another major risk factor for MAFLD in both adults and children since it favors fat accumulation within the liver by decreasing the secretion of VLDL-mediated lipids [39,40]. Several other gene variants were also related to an increased risk for MAFLD in adults, among which were the glucokinase regulator, membrane bound O-acyl transferase 7, Mer tyrosine kinase, interferon-λ4, 17-β hydroxysteroid dehydrogenase 13, and apolipoprotein B [41,42,43,44,45], but further studies are required to determine their effect on MAFLD development in children. Contrariwise, a variant of the gene encoding protein phosphatase 1 regulatory subunit 3 was proved to have a protective effect on MAFLD development, since it was related to a shift in substrate utilization in the hepatocytes from de novo lipogenesis to glycogen synthesis [41,46,47].

Another potential mechanism that contributes to the development of progressive MAFLD is represented by telomere shortening that is followed by early cell senescence [48]. Thus, mutations in the telomerase reverse transcriptase gene were related to the development and progression of this condition [49]. Additionally, it is of major importance to promptly recognize certain rare genetic disorders that can present as MAFLD in children and young adults, such as lysosomal acid lipase deficiency [50].

Recent evidence has suggested that the microbiome is a crucial contributor in the pathogenesis of MAFLD. Thus, animal-model studies, which inoculated germ-free mice with feces from obese mice with hyperglycemia, found that the first group also developed hyperglycemia and hepatic steatosis after inoculation [51]. In the setting of obesity, bacterial overgrowth and intestinal dysbiosis favor the translocation of bacterial products like lipopolysaccharides and the bacterial production of alcohol, which together promote liver inflammation [52]. The aforementioned lipopolysaccharides have the ability to activate Toll-like receptors, mainly TLR4, resulting in an abundant secretion of inflammatory cytokines, such as IL6, TNF and IL1β, with a dichotomous role, causing an increase in liver inflammation and worsening of insulin resistance [53]. Moreover, studies that assessed the bacterial phyla in mice and individuals with obesity showed an increase in Firmicutes, along with a decrease in Bacteroidetes phyla, as a response to obesity due to the dietary factors that are clearly related to microbial dysbiosis in the setting of this nutritional imbalance [54,55]. These processes are maintained by portal hypertension, which develops as the liver disease progresses, and will act as a supplementary factor in allowing the translocation of bacterial products through the promotion of gut permeability [55]. The intestinal barrier is a complex structure that includes desmosomes, adherens, and tight junctions. It serves as a network that mechanically binds the adjacent enterocytes, being able to seal the intercellular space, the Paneth cells, and the mucus layer coating the cell surface [56]. Its normal functioning is critical for hindering the passage of several food-derived and bacterial products into the portal blood flow, but if intestinal barrier function is altered, these compounds will enter the portal circulation and result in an increase of TLRs activation, enhancing the local inflammatory and fibrogenic responses [57]. Multiple studies proved that subjects with MAFLD associate an impairment of intestinal permeability, which was proved to be more severe in patients with NASH, suggesting that the degree of gut barrier integrity disruption might modulate liver injury and inflammation [58,59]. The down-regulation of the major tight junction proteins was underlined as a potential mechanism for the disruption of gut barrier integrity in patients with MAFLD [60]. Furthermore, it was proved that obesity, the most frequent comorbidity of MAFLD, is associated with important changes in the intestinal barrier’s function [61].

## 4. Diagnosis

### 4.1. Risk Factors

Apart from being overweight and obese, which are strongly related to the development of MAFLD in children, the male gender has been proved to be one of the most important risk factors for NAFLD in both children and adults. Thus, NAFLD prevalence was proved to be higher in the overweight and obese prepubertal male children, in comparison to normal-weight age-matched pairs, but it was also higher in males when compared to the age-matched females of the same BMI [13,62]. The gender differences might be explained by the liver-protective role of estrogens in females, but also by the well-documented negative role of androgens in aggravating MAFLD [63,64]. Nevertheless, variation in MAFLD rates were noticed in children of different races, revealing that being of Hispanic race is a risk factor, while being of a black race seems to have a protective effect [13,62,65,66]. In addition, children that originate from families with obesity, MAFLD, insulin resistance, and type 2 diabetes mellitus should be screened for MAFLD [67].

Low-birth weight is associated with an early catch-up growth, and it increases the risk for early obesity and subsequent development of MAFLD [68]. In terms of the dietary factors, consumption of fructose-enriched drinks is associated with MAFLD—while breast-feeding might decrease this risk [69,70]. Individuals with obstructive sleep apnea should also benefit from a screening for MAFLD since it was proved that this condition strongly suggests insulin-resistant MAFLD [71]. Polycystic ovarium syndrome, with its increased levels of androgens causing insulin resistance, might also be defined as a risk factor for MAFLD [72].

### 4.2. Clinical Features

Usually, pediatric patients with MAFLD have nonspecific symptoms consisting of fatigue, malaise, or diffuse abdominal pain, especially in the upper right quadrant, which might be associated with an advanced degree of fibrosis [13]. Hepatomegaly is a common sign of MAFLD, being detected in up to 50% of the cases [62]. Acanthosis nigricans is a typical sign of hyperinsulinemia, which has been noticed in up to a half of the children with biopsy-proved MAFLD [67].

### 4.3. Laboratory Tests

The development and validation of novel non-invasive biomarkers for the diagnosis of pediatric MAFLD is an essential current topic of research. Currently there are two types of proposed biomarkers: tests for the diagnosis of MAFLD and MASH, and tests meant to quantify the degree of fibrosis.

Despite its imperfectness, ALT is the most commonly used test for the diagnosis of MAFLD, since it was proved to be strongly correlated with the presence of steatosis [73]. Previous studies proved that children who were overweight/obese had significantly higher levels of ALT when compared to the normal-weight children [74,75]. The evidence in children remains scarce in terms of the usefulness of ALT for establishing this diagnosis, and the existing data in children with obesity are contradictory, due to the wide differences in the used ALT cutoffs and reference standards [76,77,78,79]. Nevertheless, Schwimmer and Radetti used a comparable cutoff, but their findings were not similar. Thus, Schwimmer proved that this test displayed a sensitivity of 88% and a specificity of 26% [73], whilst Radetti reported a sensitivity of only 54%, but a specificity of up to 100% [78]. It is clear that the thresholds for ALT when used as a screening tool for steatosis in children with MAFLD are far from being established, but according to the NASPGHAN guideline, a persistently elevated value of ALT >2x ULN is normal (52 IU/L in male children and 44 IU/L in girls) and might be used in order to select the patients that require further evaluation [80]. Other laboratory biomarkers, such as aspartate-amino transferase (AST), the AST/ALT ratio (AAR), the AST/platelet ratio (APRI), gamma-glutamyl transferase (GGT), bilirubin, glucose, insulin, triglycerides, the Homeostatic Model Assessment for Insulin Resistance (HOMA-IR), alpha2-macroglobulin, apolipoprotein A1, haptoglobin, leptin, interleukin-6 (IL-6), tumor necrosis factor (TNF) α, and fibroblast growth factor-21 (FGF-21) were also shown to be correlated with the presence of MAFLD [2,81,82]. Thus, studies performed on children with obesity proved that both AAR and APRI are significantly associated with the presence of obesity but failed in identifying a correlation with elastography values [75,83]. Moreover, a recent study that compared overweight/obese and normal-weight children indicated that the first group presented significantly higher levels of triglycerides, leptin, IL-6, and TNF α [82]. Nevertheless, their usefulness in clinical practice remains limited due to the small sample size, lack of validation, and proper comparison to currently used biomarkers (ALT) or ultrasound [84,85].

The Pediatric NAFLD Fibrosis Index (PNFI) was designed for the quantification of fibrosis in children and it results from three measurements: age, waist circumference and triglyceride levels [86]. According to Nobili et al., PNFI can be calculated following 2 steps: (1) the calculation of the linear predictor: *lp* = −6.539 × log_e_ [age (years)] + 0.207 × waist (cm) + 1.957 × log_e_ [triglycerides (mg/dl)] − 10.074; and (2) the transformation of the linear predictor into the PNFI: PNFI = 10/1 + e^−*lp*^ [86]. Despite its low cost and the strong positive predictive value for diagnosing fibrosis, its lack of significant negative prediction limits its utility in practice [45]. Nevertheless, its usefulness is augmented if combined with the enhanced liver fibrosis (ELF) test, which assesses a score-based tissue inhibitor of metalloproteinases 1, hyaluronic acid, and amino-terminal propeptide of the type III procollagen [87]. Studies proved that a value of PNFI >9 is strongly associated with the presence of fibrosis in children with MAFLD, whereas a value <3 is reliable for its exclusion. In addition, a value of ELF >8.49 was indicative of fibrosis in 97% of the children with this condition [45]. In setting the PNFI value between 3.47–8.99, it is recommendable to use the combined PNFI and ELF tests in order to increase the prediction of the fibrosis diagnosis, being proved that this approach is predictable in up to 86.4% of the children with MAFLD-associated fibrosis [87]. This combined approach, if used as a first-line screening test, might hinder the progression of fibrosis towards severe liver damage—i.e., fibrosis stage 3 (F3), F4, or cirrhosis [45].

### 4.4. Imaging

Ultrasonography is the most frequently used tool for the detection of fibrosis due to its low costs, wide availability, and user-friendly pattern [45]. Several ultrasonographic features such as liver echogenicity, hepato-renal echo contrast, and visualization of the hepatic vessels, liver parenchyma, and diaphragm, were found to accurately estimate the degree of liver steatosis with a sensitivity of 79.7% and specificity of 86.2% for diagnosing moderate-to-severe steatosis in children with MAFLD [88]. Nevertheless, the sensitivity of the ultrasound is low if the fat accumulation impairs <30% of the liver parenchyma or if the BMI is >40 kg/m^2^ [45]. Another well-acknowledged limitation of ultrasonography is that it cannot rule out the presence of either NASH or fibrosis. Taking into account these factors, along with the differences related to the operator variability, hepatic ultrasonography results should be interpreted with deep caution [89]. The ESPGHAN recommends the use of ultrasonography for children with obesity, signs of insulin resistance, and/or hyperinsulinemia [1].

Due to these inconveniences, the assessment of computer tomography (CT) and magnetic resonance imaging (MRI)-based techniques revealed a higher accuracy for the quantification of steatosis as compared to ultrasonography [45]. Thus, CT enables quantitative evaluation of liver steatosis since the beam attenuation in the liver is related to liver fat contents, but it has been replaced by MRI—a more accurate technique for detecting steatosis—which is also free of ionizing radiation [2]. Furthermore, the MRI proton density fat fraction (MRI-PDFF) technique was found to have an even higher diagnostic accuracy for classifying the degree of histological steatosis and predicting histological steatosis in children with MAFLD [90], but it is currently not enough to replace liver biopsy in children. Schwimmer et al. assessed the accuracy of both MRI and MRI-PDFF in comparison to liver biopsy in children with MAFLD and identified an overall accuracy of MRI-PDFF for predicting histologically-confirmed steatosis of 56%, and an AUROC of 0.82 in distinguishing grade 0 steatosis from grade 1 [91]. Moreover, the authors found a mean MRI-PDFF of 2.6% for grade 0 steatosis, 9.2% for grade 1, 15.1% for grade 2, and 26.8% for grade 3. Another essential advantage of MRI-PDFF is represented by its ability to map the entire liver for an accurate measurement of the degree of hepatic steatosis, even in the setting of uneven fatty change [2].

Elastography-based methods were developed for the quantification of liver fibrosis. Transient elastography (TE) is one of the most accurate, non-invasive, ultrasound-based methods in terms of detecting clinically significant liver fibrosis, advanced fibrosis, and cirrhosis—being validated for a wide spectrum of chronic liver diseases [92]. The probe consists of a small vibrating transducer, which produces a shear wave, being attached to an ultrasound probe, which measures its propagation. It allows the measurement of 1/500 of the liver, being less prone to sampling error in comparison to a liver biopsy [2]. Most of the studies performed in adults with MAFLD proved that this method is excellent for ruling out the presence of fibrosis, as well as differentiating between both F3/F4 and milder stages of fibrosis—but it only has 78% accuracy for diagnosing ≥F2 fibrosis [83]. Moreover, a recent study performed on children with obesity indicated that liver stiffness values measured from TE were significantly higher in children with obesity when compared to normal-weight ones [75]. Similar findings were reported by Cho et al. who stated that TE is a reliable method for the screening of steatohepatitis and liver fibrosis in Japanese children with obesity [93]. Another study performed on pediatric patients proved that liver stiffness values ranging between 7–9 kPa were predictive for fibrosis stages 1 or 2, whilst a value of at least 9 was associated with advanced fibrosis [94]. Studies performed on children with biopsy-proved MAFLD concluded that TE has great accuracy for predicting the presence of liver fibrosis, irrespective of its severity [94,95]. Similarly, two-dimensional shear wave-elastography and acoustic radiation force impulse represent another two ultrasound-based tools that proved to be useful in diagnosing steatohepatitis and liver fibrosis in children with obesity [75,96].

### 4.5. Liver Biopsy

Liver biopsy remains the most accurate method for both grading and staging of MAFLD, but its utility, especially in pediatric ages, is limited due to its potential related complications such as pain, internal bleeding, pneumothorax, leak of bile from the gallbladder or liver, and being subjected to errors due to the small area of the organ sampled [45,97]. Despite these risks, the NASPGHAN guidelines recommend liver biopsy in patients with increased levels of ALT (>80 U/L), AAR >1, or splenomegaly, altogether defining a high risk of MASH and/or advanced fibrosis [80]. Several key features were defined as diagnostic and staging characteristics for MAFLD in adults, like steatosis, hepatocyte ballooning, inflammation and fibrosis. Nevertheless, these features often differ in children [65,98], having been proved that most of these patients are found with overlapping features of NASH type 1 and 2 [99,100]. Studies that aimed to find an explanation for these differences found that genetic background, along with the activation of the Hedgehog signaling pathway, might be incriminated in triggering these variations between children of different ethnicities, as well as between the two types of NASH [45,101].

The most important scoring system for the histological grading of MAFLD in pediatric patients is the NAFLD activity score (NAS), since it assesses the spectrum of histological patterns specific to this condition [102]. Taking into account the aforementioned histological differences between adults and children, the Pediatric NAFLD Histological score was proposed, but its usefulness is mainly limited to clinical research rather than clinical practice [103].

## 5. Treatment

Considering that obesity is the main cause of MAFLD in children, lifestyle intervention represents the first line in the therapeutic approach for these patients. Therefore, dietary changes, behavioral modifications and physical exercise remain the crucial components of lifestyle interventions in children with MAFLD. Furthermore, it was proved that weight loss of more than 7–10% has the ability to reverse MAFLD in most of the patients [104,105,106], but unfortunately the withdrawal rate during intervention is usually high and only a small percentage of patients achieve this weight loss [104,107,108]. The few studies that assessed the effects of the lifestyle interventions on inflammation and fibrosis proved that after at least 12 months, the children that adhere to these programs were found with considerable improvements in both inflammations [108] and fibrosis [109]. Moreover, exercise alone might also be associated with a reduction of intrahepatic fat measure by MRI in children with MAFLD, but unfortunately it has no impact on ALT levels [110,111].

In terms of pharmacological treatment, there are no currently approved guidelines in adults and children with MAFLD. Still, the 2018 American NAFLD guidelines reached the consensus that vitamin E might be beneficial in children with NASH [112] since it was shown to improve steatosis and inflammation, but not fibrosis [113,114]. Therefore, α-tocopherol or vitamin E was proved to dampen the impact of cell injuries on the surrounding healthy liver tissue [115]. Based on its antioxidant properties, vitamin E might alleviate oxidative stress during fulminant cell death [114,116]. Pioglitazone was also proved to be beneficial in reversing NASH and improving fibrosis in both non-diabetic and diabetic patients [114,117]. The antioxidant cysteamine bitartrate, probiotics, docosahexanoic acid, fish oil, ursodeoxycholic acid, carnitine, and insulin oral sensitizers were also assessed in children with MAFLD, but they either showed little effectiveness, or none in comparison to the lifestyle treatment alone [118,119,120,121,122,123]. The modulation of the glucagon-like-1 (GLP-1) incretin pathway proved to be extremely useful in the management of patients with type 2 diabetes mellitus due to its several positive effects in modulating body metabolism [124]. Thus, GLP-1 analogues, i.e., liraglutide and semaglutide, seem to be promising in targeting the altered metabolism in MAFLD [125,126,127,128] patients, resulting in the histological resolution of MASH [129].

Several clinical trials are ongoing in children, studying the effects of multiple drugs such as the monoclonal antibody anti-LPS, the synthetic analogue of leptin—metreleptin, the aminothiol salt—cysteamine bitartrate, and losartan [2,130,131].

The differences and similarities between pediatric and adult MAFLD are highlighted in Table 1.

We highlighted the key points of this review in Table 2.

## 6. Conclusions

MAFLD represents an emerging global public health problem in pediatric patients, mostly due to the alarmingly increased incidence. Despite the efforts from the last decade to identify accurate, non-invasive, and widely available tools for both screening and diagnosis of steatosis, inflammation, and fibrosis that can replace liver biopsy, the evidence in pediatric patients remains scarce. Moreover, there is a crucial need for further studies in order to establish the proper protocols for screening, diagnosis, and treatment in children with MAFLD.

## Figures and Tables

**Table 1:** Differences and similarities between pediatric and adult MAFLD.

	Differences		Similarities
	Children	Adults	Children & Adults
Epidemiology	Prevalence in USA −9.6% [13] Prevalence increases with age among obese/overweight children (17.3–38%) [13] Risk factors: PCOS [132], obstructive sleep apnea and hypoxemia [133], psoriasis [134], and pan-hypopituitarism [135], genetic and environmental factors [136]	Prevalence in western countries is 20–30% [10] and 5–18% in Asia [8,137] Type 2 diabetes—high risk for NAFLD (45–75%) [138,139] Older age, male gender [138,139] major risk factors	Risk factor—obesity/overweight [140] Hispanic ethnicity risk factor [13]
Diagnosis	AST/ALT ratio, NAFLD fibrosis score (NFS), APRI, FIB4-score—not accurate in predicting fibrosis [141,142]	AST/ALT ratio, NAFLD fibrosis score (NFS), APRI, FIB4-score—accurate in predicting fibrosis [141,142]	Liver biopsy—gold standard [139,143] Noninvasive diagnosis of steatosis & fibrosis—useful in clinical practice [143] ultrasonography & liver function test is used to screen the liver function in obese children [144] Analysis of breath volatile organic compounds as a MRS [145,146] Transient elastography, shear wave elastography, Fibroscan, MR elastography [75]
Histology	Steatosis: periportal zone 1 or azonal distribution, typically moderate to severe [65,98] Inflammation: portal inflammation—common [65,98] Ballooning—Mallory’s hyaline bodies are with low frequency & hepatocyte ballooning is rare [65,98] Portal-periportal fibrosis [65,98]	Steatosis: perivenular zone (acinar zone 3), typically mild to moderate [65] Inflammation: lobular inflammation—common [65,98] Ballooning degeneration -common [65,98] Perisinusoidal fibrosis [65,98]	–
Molecular markers	–	–	Fecal—gut microbiota dysbiosis [52,147] Circulating—Adipocytokines and Hepatokines [98,100,148] Tissue specific—Macrophage activation- Activation of hepatic progenitors [98,100,148]
Genetic variants PNPLA3 [31,149] GCKR [150] APOC3 [151] LPINI [152]	Associated with MAFLD, association with NASH unclear [31,149] – – inverse association with NASH [152]	Strongly associated with MAFLD and NASH [31,149] – – not investigated [152]	– Associated with NASH [150] No correlation with MAFLD [151] –
Management Lifestyle modifications	–	–	Change in lifestyle, gradual weight reduction, physical exercise—mainstay of treatment for MAFLD, improving the biochemical parameters & liver histology [16,108,139]
Bariatric surgery	No studies [153,154]	Significant improvement in histology after bariatric surgery [114,154]	–
Pharmacotherapy Metformin Vitamin E Omega-3 Fatty acids Obeticholic acid	–	–	Metformin 2 × 500 mg/day—no benefit to children & adults with MAFLD, nor improvement of ALT [74] nor resolution of NASH [113,139,155,156,157]
	–	–	Vitamin E 800 IU/day improves histology in children & adults with NASH, without reduction of ALT levels [113,155,156].
	DHA 250 mg/day or 500 mg/day improves liver steatosis in children [139,158,159]	EPA-E—no effect on the histology in NASH [158,160]	–
	Not available data data [161,162]	Improves NASH, hepatocellular ballooning, lobular inflammation and fibrosis [161,162]	–
Outcome	Few data on the prognosis and clinical complications of MAFLD due to low number of studies in children [163] Rare incidence of HCC [98] Incidence of cirrhosis 1–2% [98]	More data in adults—higher number of studies [163] Significant risk of developing HCC [164] Incidence of cirrhosis 5–10% [98]	Progression to fibrosis, NASH and cirrhosis [163] Metabolic syndrome (obesity, type 2 diabetes, dyslipidemia, and/or hyperglycemia)—29–83% [139] & cardiovascular disease [98]

Legend: ALT—Alanine aminotransferase; APOC3—Apolipoprotein C3; APRI—AST/platelet ratio index; AST—Aspartataminotransferase; FIB4-score—fibrosis 4 index; DHA—Docosahexaenoic acid; EPA-E—Eicosapentaenoic acid; HCC—hepatocellular carcinoma; GCKR—Glucokinase Regulator; MRS—magnetic resonance spectroscopy; LPINI—Protein Coding Lipin 1; MAFLD—metabolic associatec fatty liver disease; NAFLD—non-alchoolic fatty liver disease; NASH—nonalcoholic steatohepatitis; NFS—NAFLD fibrosis score; PCOS—polycystic ovarian syndrome; PNPLA3—Patatin-like phospholipase domain-containing protein 3.

**Table 2:** Key points of MAFLD in children.

Parameters	Key Points
Epidemiology	Prevalence—3–10% in general pediatric & increase of up 70% in individuals with metabolic comorbidities Higher prevalence in children with obesity Affects predominantly males and Hispanics of Indigenous American ↑ALT levels ranging between 8–42% Ultrasonography—“bright” liver 1.7 to 77% Histological MAFLD ranging from 0.7% in 2–4-year-old to 17.3% in 15–19-year-old & up to 38% in children with obesity
Risk factors for MAFLD	Overweight and obesity Male gender Hispanic race Family history of obesity, MAFLD, insulin resistance, and type 2 diabetes mellitus Low-birth weight Consumption of fructose-enriched drinks Obstructive sleep apnea PCOS PNPLA3 gene
Clinical features	Fatigue Malaise Diffuse abdominal pain especially in the right upper quadrant Hepatomegaly—up to 50% of the cases Acanthosis nigricans—sign of hyperinsulinemia
Laboratory tests	ALT↑ > 2x ULN normal, AST AAR, APRI GGT, bilirubin, Glucose, insulin Triglycerides HOMA-IR Alpha2-macroglobulin, apolipoprotein A1, haptoglobin, Leptin, IL-6, TNF α, FGF-21 PNFI > 9 is strongly associated with the presence of fibrosis in children ELF test > 8.49 was indicative for fibrosis in 97% of the children
Imaging	Ultrasonography: liver echogenicity, hepato-renal echo contrast, and visualization of hepatic vessels—fairly estimate the degree of liver steatosis children with obesity, signs of insulin resistance and/or hyperinsulinemia CT & MRI—higher accuracy for the quantification of steatosis MRI-PDFF—higher accuracy for classifying histological steatosis degree TE—most accurate non-invasive ultrasound-based method for detecting liver fibrosis
Liver biopsy	The most accurate diagnosis for MAFLD Histological aspect: Steatosis: periportal zone 1, portal inflammation, periportal fibrosis
Treatment	Dietary changes and physical exercise—crucial components Pharmacological treatment—none approved Vitamin E is benefic in children—to improve steatosis and inflammation, but not fibrosis Pioglitazone—beneficial in reversing NASH and improving fibrosis in non-diabetic and diabetic patients Cysteamine bitartrate, probiotics, DHA, fish oil, ursodeoxycholic acid, carnitine and insulin oral sensitizers—low efficacity GLP-1—useful in the management of patients with type 2 diabetes mellitus

Legend: AAR—AST/ALT ratio; ALT—Alanine aminotransferase; APRI—AST/platelet ratio index; AST—Aspartataminotransferase; CT—computer tomography; DHA—Docosahexaenoic acid; ELF—enhanced liver fibrosis test; FGF-21—fibroblast growth factor-21; GGT—gamma-glutamyl transferase; GLP-1—glucagon-like-1; HOMA-IR—Homeostatic Model Assessment for Insulin Resistance; IL—6—interleukin-6; MRI—magnetic resonance; MRI-PDFF—MRI proton density fat fraction; MAFLD—metabolic associated fatty liver disease; NASH—nonalcoholic steatohepatitis; PCOS—polycystic ovarian syndrome; PNFI—The Pediatric NAFLD Fibrosis Index; PNPLA3—Patatin-like phospholipase domain-containing protein 3; TE—Transient elastography; TNF α—tumor necrosis factor.

## Data Availability

Not applicable.
